# Functional characterization of *UL50* gene reveals its essential role in duck enteritis virus replication and pathogenesis

**DOI:** 10.1080/01652176.2026.2649575

**Published:** 2026-03-24

**Authors:** Su-xin Huo, Liu Chen, En-dong Bao, Zheng Ni, Wei-cheng Ye, Jiong-gang Hua, Tao Yun, Yuan Fu, Yue Wu, Fang-zhou Ding, Xu Gao, Rui Zhong, Zong-xiao Wang, Cun Zhang, Yin-chu Zhu

**Affiliations:** a State Key Laboratory for Quality and Safety of Agro-Products, Zhejiang Key Laboratory of Livestock and Poultry Biotech Breeding, Key Laboratory of Livestock and Poultry Resources (Poultry) Evaluation and Utilization, Ministry of Agriculture and Rural Affairs, Zhejiang Engineering Research Center for Poultry Breeding Industry and Green Farming Technology, Institute of Animal Husbandry and Veterinary Sciences, Zhejiang Academy of Agricultural Sciences, Hangzhou, People's Republic of China; b College of Veterinary Medicine, Nanjing Agricultural University, Nanjing, People's Republic of China; c College of Veterinary Medicine, Inner Mongolia Agricultural University, Hohhot, People's Republic of China

**Keywords:** *UL50* gene, gene deletion, replication efficiency, virulence attenuation, neuronal infection, pathogenicity, duck enteritis virus, α-herpesvirus

## Abstract

Duck plague, caused by a highly contagious *α*-herpesvirus, poses a major threat to waterfowl farming. Although *UL50* homologs have been studied in mammalian *α*-herpesviruses such as HSV-1 and PRV, their role in avian herpesviruses remains unknown. Here, we investigated the function of the DEV *UL50* gene, which encodes a conserved viral dUTPase, using bioinformatics, molecular biology, and virological approaches. Sequence analysis confirmed that DEV *UL50* retains conserved catalytic motifs and structural features characteristic of *α*-herpesvirus homologs. A *UL50*-deleted mutant (ΔUL50) was constructed using the Red recombination system. *In vitro*, ΔUL50 exhibited reduced replication efficiency in DEFs, characterized by smaller plaques and lower viral titers, although overall growth kinetics were broadly similar to the WT. Notably, in duck DRG neurons, ΔUL50 replication was nearly abolished. GFP-reporter BAC viruses further confirmed that ΔUL50 failed to spread in DRG neurons but retained propagation in DEFs, indicating a cell-type–dependent replication defect. *In vivo*, ΔUL50 displayed markedly reduced viral loads and attenuated virulence, with no mortality and milder clinical and histopathological changes. These findings demonstrate that *UL50* is dispensable but replication-supportive, particularly in non-dividing neuronal cells, highlighting its role in DEV pathogenicity and extending understanding of *α*-herpesvirus biology beyond mammalian systems.

## 1. Introduction

Duck plague, caused by duck enteritis virus (DEV), is a highly contagious and acute disease first officially reported in 1923 (Baudet 1923). Despite nearly a century of research, it remains difficult to eradicate. Its persistence is attributed to strong environmental resistance, diverse transmission pathways, latent carriers, and the role of wild waterfowl as reservoirs. Susceptible animals exhibit a high infection rate, with mortality reaching up to 100% in severe outbreaks, leading to significant economic losses in the global poultry industry. Although vaccination with inactivated or attenuated vaccines remains the primary control strategy (Yang et al. 2010, 2017), outbreaks continue to occur. Since 2023, the disease has been reported across multiple regions of China, affecting breeder ducks, commercial meat ducks, Muscovy ducks, and layer ducks, with some cases even involving breeding and laying geese. Research into DEV pathogenicity remains limited, restricting advancements in control strategies. The DEV genome encodes numerous proteins with unknown functions, some of which may play important roles in regulating virulence and host responses. Further exploration of viral pathogenic mechanisms in waterfowl is essential, especially considering their distinct immune system and nucleotide metabolism compared with mammals, which may underlie unique host–virus interactions.

DEV is classified within the genus *Mardivirus* and the subfamily *Alphaherpesvirinae* of the family *Herpesviridae* (Chen et al. 2013). Structurally, it features linear double-stranded DNA, an icosahedral capsid, an amorphous tegument, and a bilayer lipid envelope, reflecting a complex viral architecture. Approximately 78 open reading frames (ORFs) have been predicted, encoding structural and non-structural proteins that collectively regulate viral replication and virulence (Tang et al. 2017). The advent of technologies like bacterial artificial chromosome (BAC) systems and CRISPR have significantly accelerated herpesvirus research, driving notable advances in the molecular biology studies of DEV. Several DEV virulence genes have been identified, including *UL23* (Li et al. 2006), *US10* (Zheng 2020), *LORF5* (Shen et al. 2021), *US8* (Ning et al. 2022), *US3* (Wei 2023), and *LORF3* (Shen et al. 2023). Nevertheless, many ORFs remain insufficiently characterised. One such gene is *UL50*, located within the unique long (UL) region and annotated to encode a conserved dUTPase. In mammalian *α*-herpesviruses such as pseudorabies virus (PRV) and herpes simplex virus type 1 (HSV-1), *UL50* homologues have been shown to influence replication efficiency and virulence in a context-dependent manner (Pyles et al. 1992; Jons et al. 1997). However, their functional roles in avian *α*-herpesviruses remain largely unknown. Members of the *α*-herpesvirus subfamily are well known for their neurotropic properties and their ability to infect and replicate within neuronal tissues. Unlike proliferating fibroblasts, neuronal cells are terminally differentiated and non-dividing, possessing limited nucleotide biosynthesis capacity. These metabolic constraints suggest that viral factors involved in nucleotide metabolism may play particularly important roles in supporting viral DNA replication within neuronal environments.

Deoxyuridine triphosphate (dUTP) is an intermediate generated during pyrimidine nucleotide metabolism, and its intracellular concentration is tightly controlled to prevent uracil misincorporation into DNA (Gadsden et al. 1993; Palinkas et al. 2019). Nucleotide pools differ substantially between proliferating and terminally differentiated cells, with non-dividing cells such as neurons exhibiting reduced nucleotide biosynthetic activity (Hizi and Herzig 2015). Such differences are particularly relevant for DNA viruses that depend on host nucleotide availability. In several mammalian *α*-herpesviruses, the deletion of viral dUTPase has been reported to affect viral replication or virulence in a host- and cell type–dependent manner. Owing to interspecies differences in immune regulation and nucleotide metabolism, however, findings from mammalian systems may not fully reflect *UL50* function in avian hosts. Whether *UL50* fulfills conserved or distinct roles in waterfowl therefore remains to be determined.

In this study, we investigated the biological role of the DEV *UL50* gene using molecular genetics, cellular infection models, and *in vivo* experiments in ducks. A *UL50*-deleted mutant (ΔUL50) was generated using a two-step Red recombination system based on the a previously constructed infectious DEV XJ BAC clone (Huo et al. 2024). We first assessed viral replication in duck embryo fibroblasts (DEFs), followed by evaluation of viral distribution and pathogenicity in experimentally infected ducks. Notably, reduced viral loads in brain tissues prompted further analysis of replication in primary duck dorsal root ganglion (DRG) neurons. By comparing parental, ΔUL50, and revertant viruses in dividing and non-dividing cells, we provide a systematic assessment of *UL50* function in DEV replication and pathogenicity in its natural avian host.

## 2. Materials and methods

### 2.1. Cells, virus, and primers

Duck embryo fibroblasts (DEFs) were prepared from 12-day-old specific-pathogen-free (SPF) embryonated eggs and cultured in Dulbecco’s modified Eagle medium (DMEM; Thermo Fisher Scientific, USA), supplemented with 10% foetal bovine serum (FBS; Thermo Fisher Scientific, USA), at 37 °C in 5% CO_2_.

The DEV vaccine strain (C-KCE strain) and the DEV standard highly virulent strain (CV strain) used in this study were preserved in our laboratory. Wild-type DEV XJ (GenBank accession NO. OR757570), originally isolated in 2021 (Huo et al. 2024) and maintained in our laboratory, was used for bacterial artificial chromosome (BAC) construction and subsequent genetic manipulation. The DEV XJ *UL50*-deleted mutant strain (here referred to as ΔUL50) and DEV XJ *UL50* reverting mutant strain (here referred to as RΔUL50) were constructed using the two-step Red recombination system based on the bacterial artificial chromosome of DEV XJ gene-editing platform constructed in our laboratory. All primer sequences and products are shown in Table 1.

**Table 1.** Primers used in this article.

Primers	Sequence (5'–3')	Product
UL50-ΔF	AAAAAGACGTGCGATTTAATGTTTTGTGATCTGTTTGACAGGCTTCTATGGAGACAGCTTGGATGACGACGATAAGTAGGGA	ΔUL50 targeting fragment
UL50-ΔR	GGAGGGTGTAGCCGGCACAGAAGCTGTCTCCATAGAAGCCTGTCAAACAGATCACAAAACGGGTAATGCCAGTGTTACAAC	
RUL50-F	AGAGGAGGGTGTAGCCGGCACAGAAGCTGTCTCCATAGAAGCCATGGCAACTGGAAATTGTGGAGC	UL50-returned targeting fragment
RUL50-R	AAAACTAAAAAGACGTGCGATTTAATGTTTTGTGATCTGTTTGACATTATATCCCAGTTGATCCGA	
RUL50-Kan-F	CGAAACCTCCTTCTCTTCTCTCGCTTGTCATAGCCTCCGTGTCGTAGTGAAGGGTGAAACGCCAAACCGGTGAAGGATGACGACGATAAGTAGGG	UL50-returned targeting fragment
RUL50-Kan-R	CCGCCTGCCATGCTGTCGACAAGCGCTCCGTATGTAACTCACCGGTTTGGCGTTTCACCCTTCACTACGACACGGAGGCTATGCAACCAATTAACCAATTCTGATTAG	
UL50-JD-F	AAGGAGAAACCCGAAGAG	UL50 deletion identified
UL50-JD-R	GAGAAGGCAACTGACACG	
qUL44-F	GGTTCGCTTCTTCTCGGAAGT	UL44
qUL44-R	TCCCCTTCGCCATTCCATA	
UL44-Probe	CAGAGATCACATGTAAAGC	
qUL50-F	ACCTCCTTCTCTTCTCTC	UL50
qUL50-R	CCCACCACAACTATTCAG	
UL50-Probe	CATAGCCTCCGTGTCGTAGTGAA	

### 2.2. Plasmid and antibodies

GS1783-BAC-XJ, pCAGGS-NLS/Cre, pET28a(+) and pLAY2KAN carrying kanamycin resistance gene and I-Sce I were provided by our laboratory. The rabbit polyclonal antibodies of *UL50* protein were generated in this study. The HRP-labelled Goat Anti-Rabbit IgG (H + L) and the DYKDDDDK tag Rabbit PolyAb were purchased from Proteintech Group, Inc. The Cy3-labelled Goat Anti-Rabbit IgG (H + L) were purchased from Beyotime Institute of Biotechnology.

### 2.3. Bioinformatics analysis of *UL50*

The amino acid sequence of DEV *UL50* protein (GenBank accession: WOZ29838.1) was retrieved from NCBI. Sequence alignment and characterisation were performed using Clustal Omega (https://www.ebi.ac.uk/jdispatcher/msa). Phosphorylation sites and signal peptides were predicted using NetPhos 3.1 (https://services.healthtech.dtu.dk/services/NetPhos-3.1/) and SignalP 6.0 (https://services.healthtech.dtu.dk/services/SignalP-6.0/), respectively. Subcellular localisation was assessed using DeepLoc (https://services.healthtech.dtu.dk/services/DeepLoc-2.1/). The three-dimensional structure of the *UL50* protein was predicted using the AlphaFold2 server (https://colab.research.google.com/github/sokrypton/ColabFold/blob/main/AlphaFold2.ipynb). The protein structure superimposition was performed using PyMOL version 2.6, and the root-mean-square deviation (RMSD) values were calculated and provided by the PyMOL software.

### 2.4. Construction of recombinant viruses

A full-length *UL50* open reading frame-deleted virus (ΔUL50 strain) and its repaired strain (RUL50 strain) were constructed using a two-step Red-mediated recombination approach. The targeted fragments for the ΔUL50 strain and RUL50 strain were amplified using primers UL50-ΔF/R, RUL50-F/R, and RUL50-Kan-F/R. These fragments were electroporated into *E. coli* GS1783-BAC-XJ competent cells. In the first step of homologous recombination, facilitated by homologous recombinase, the target fragments were integrated into the viral genome within the BAC. In the second step, the kanamycin resistance gene was excised using I-SceI and the DNA double-strand break repair mechanism. To confirm the modifications, PCR amplification was performed with UL50-F/R primers, restriction fragment length polymorphism (RFLP) identification was conducted using *BamH*I and *Xho*I, and Western blotting was used to verify the deletion of *UL50* protein. The resulting positive BAC plasmids were transfected into DEFs using calcium phosphate precipitation, ultimately generating ΔUL50-BAC or RUL50-BAC recombinant strain. BAC genome was excised by co-transfecting pCAGGS-NLS/Cre and pBAC-ΔUL50 or pBAC-RUL50 into DEF cultures. The stable and inheritable recombinant viruses ΔUL50 and RUL50 were obtained by limiting dilution and plaque purification and then identified by PCR amplification with the primer pairs UL50-F/R and sequencing.

### 2.5. Multistep viral growth kinetics

To assess replication properties of recombinant viruses, multistep viral growth curves were performed as described previously (Huo et al. 2024), with minor modifications. DEFs in 12-well plates were infected with 0.02 MOI of indicated viruses. Cell and culture supernatant samples were collected at 0, 12, 24, 36, 48, 72, 96, and 120 hours post-infection and stored at –80 °C until the completion of sample collection. Samples were treated with a Tissuelyser-24 at 60 Hz for 90 s and centrifuged at 5000 rpm for 5 min before titration. Intracellular and supernatant viral titre were detected by determining the TCID50, and multistep growth curves were plotted based on data from three independent experiments.

### 2.6. Viral adsorption, penetration, and replication assays

**Adsorption:** DEFs cultured in 24-well plates were pre-cooled at 4 °C for 1 h. Subsequently, they were infected with the indicated viruses at an MOI of 0.1. After a 2-hour incubation at 4 °C, the cells were washed 3 times with pre-cooled PBS. The cell samples were then collected and stored at –80 °C until all sample collections were completed. **Penetration:** DEFs were infected following the adsorption procedure described above. After a 2-hour incubation at 4 °C, the cells were washed 3 times with PBS, and the medium was replaced with DMEM. The cells were then incubated at 37 °C for 1 h. To remove virions that had not entered the cells, the cells were washed with 0.05% trypsin. The cell samples were collected and stored at –80 °C. **Replication:** DEFs cultured in 24-well plates were infected with the indicated viruses at an MOI of 0.1. After a 2-hour incubation at 37 °C, the cells were washed and the medium was replaced with DMEM to continue incubation. Cell samples were collected at 6, 9, and 12 hours post-infection using the washing procedure described in the penetration section. All samples were stored at –80 °C until the completion of sample collection.

### 2.7. The plaque morphology of recombinant viruses

Plaque size assays were conducted to evaluate the cell-to-cell spread ability of recombinant viruses. DEFs in 12-well plates were infected with 0.02 MOI of indicated viruses. Following a 2-hour incubation at 37 °C, DMEM containing 1% agarose with low gelling temperature was added to cover the infected cells. After a 6-day incubation at 37 °C, the cells were fixed with 4% paraformaldehyde and stained with 1% crystal violet at room temperature. 80 randomly selected plaques were chosen for each virus and their plaque sizes were measured using ImageJ software.

### 2.8. Primary neuronal culture and neurovirulence assay *in vitro*

Dorsal root ganglia (DRG) neurons were isolated from duck embryos as previously described (Yan et al. 2019). The DRG tissues were enzymatically dissociated into single-cell suspensions and evenly seeded into 96-well plates at a uniform cell density. Neurons were cultured for 10 days before infection. Both neurons and DEFs cultured in 96-well plates were simultaneously infected with the indicated viruses at an MOI of 0.1. Samples were collected at 24, 48, 72, and 96 hours post-infection and stored at –80 °C until all time points were completed.

### 2.9. *In vivo* experiment

To investigate the potential role of *UL50* gene in the pathogenesis and lethality of DEV XJ, animal experiments were performed. 60-day-old ducks, confirmed to be free of DEV and DEV antibodies, were intramuscularly injected with 10^6^ DNA copies of liver homogenate supernatant from the parental WT (*n* = 10), ΔUL50 (*n* = 10), RUL50 (*n* = 10) and DMEM (*n* = 10) groups. The ducks were monitored daily for clinical symptoms, and those that succumbed to the disease or were humanely euthanized underwent histopathological examination. Samples of the liver, thymus, heart, brain, spleen, and intestines were collected at 1, 3, and 5 dpi from each group to evaluate *in vivo* virus replication. Additionally, the liver, intestines, and thymus were fixed in 4% paraformaldehyde for further analysis. Pathological sections were prepared by Hubei Biossci Biotechnology Co., Ltd.

### 2.10. Quantification of DEV genome copies

Total DNA strands of the supernatants were extracted using a viral genomic DNA/RNA extraction kit according to instruction (GENFINE, China). THUNDERBIRD Probe qPCR Mix (TOYOBO, China) was used to quantify viral DNA copies. The primers and probe for detecting the indicated gene by qPCR had been previously designed in our laboratory. The qPCR amplifications were carried out with the following conditions: 95 °C for 60 s, followed by 40 cycles at 95 °C for 15 s and 60°C for 45 s. The result of the qPCR were quantified by comparison with the established curve.

### 2.11. Statistical analysis

Experimental data were analysed and plotted using GraphPad Prism version 9.4. Results are presented as mean ± standard error (SE). Statistical significances difference between two groups was determined using an unpaired t-test, while comparisons among multiple groups were performed using one-way analysis of variance (ANOVA) followed by Tukey’s or Bonferroni post hoc test. All experiments were independently repeated at least three times. Statistical significances was indicated as follows: *p < 0.05, **p < 0.01, ***p < 0.001, ****p < 0.0001.

## 3. Results

### 3.1. Identification and molecular characteristics of DEV *UL50* gene

**Figure 1.**
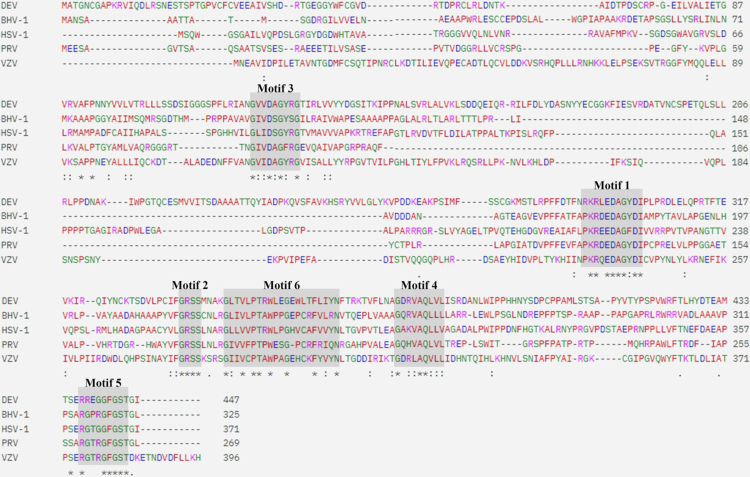
Multiple sequence alignment of *UL50-*encoded proteins. Comparison of the UL50-encoded protein from DEV with homologous proteins from BHV-1, HSV-1, PRV, and VZV. Sequences were aligned using Clustal Omega. Gaps in the alignment are indicated by dashes (‘-’); symbols ‘*’, ‘:’, and ‘.’ represent identical, conserved, and semi-conserved residues, respectively. Different colours denote different amino acid properties. Conserved motifs are highlighted with grey shading.

**Figure 2.**
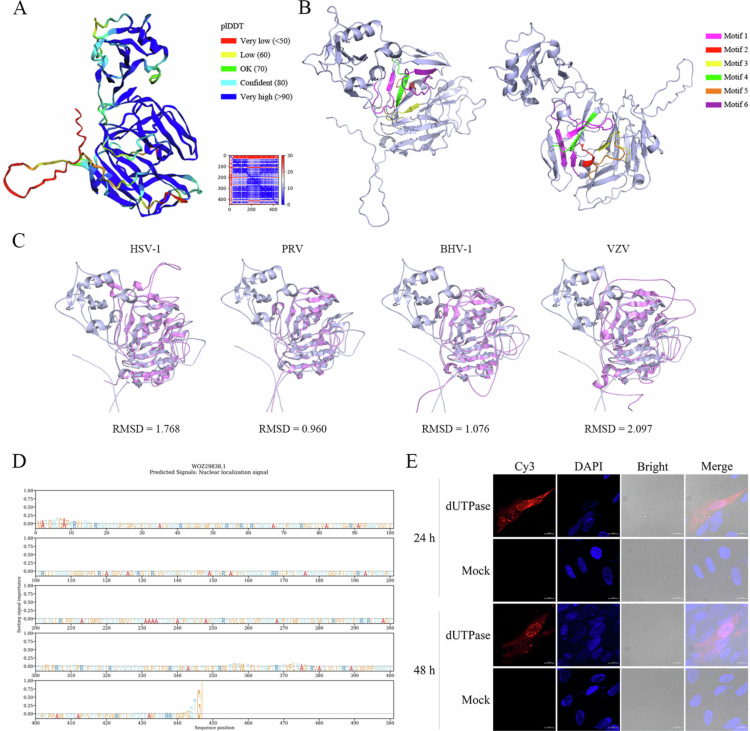
Predicted structural features and Subcellular localisation of the DEV *UL50*-encoded protein. (A) AlphaFold2 prediction of the 3D structure of the *UL50*-encoded protein, with colours indicating prediction confidence (pLDDT score): blue (very high, >90), cyan (confident, 70–90), yellow (low, 50–70), and red (very low, <50). (B) Visualisation of the six conserved motifs in the predicted structure using PyMOL, with each motif highlighted in a different colour. (C) Structure superimposition of DEV *UL50* protein (light blue) with homologous proteins from HSV-1, PRV, BHV-1, and VZV (violet), performed using PyMOL. The root mean square deviation (RMSD) values indicate structural similarity. (D) Nuclear localisation signal (NLS) prediction using DeepLoc. A prominent peak was observed around positions 445–450. (E) Subcellular localisation of DEV *UL50* protein in DEFs. Cells were either mock-transfected or transfected with pUL50-Flag. At 24 and 48 hpi, cells were fixed and stained with anti-FLAG (DYKDDDDK) rabbit polyclonal antibody followed by Cy3-conjugated goat anti-rabbit IgG. Nuclei were counterstained with DAPI (blue).

The ORF of the DEV *UL50* gene is 1344 bp in length, encoding a protein of 447 amino acids with an estimated molecular mass of 49.7 kDa and a guanine/cytosine (GC) content of 47%. Conserved domain analysis revealed the presence of five typical motifs commonly associated with the dUTPase-like protein family, together with an additional motif 6 that is characteristic of monomeric *UL50* homologues (Figure 1). These six motifs were highly conserved among *α*-herpesvirus *UL50* proteins. Multiple sequence alignment using Clustal Omega demonstrated that at least 36 amino acid residues were conserved across *UL50* homologues. To assess sequence relatedness, BLASTp analysis was performed using DEV *UL50* protein against reference sequences from the NCBI GenBank database, including BHV-1 (CAA90918.1), HSV-1 (WNK89014.1), PRV (WBR46099.1), and VZV (AAT07766.1). The DEV *UL50* protein shared sequence identities of 38.04% with BHV-1, 40.12% with HSV-1, 39.29% with PRV, and 36.81% with VZV. Structural prediction using AlphaFold2 revealed a conserved UL50-like fold with predominantly high-confidence regions (Figure 2A), and the six motifs were subsequently annotated in PyMOL (Figure 2B). Structural superimposition further demonstrated notable three-dimensional similarity between DEV *UL50* and homologous proteins from HSV-1 (RMSD = 1.768), PRV (RMSD = 0.960), BHV-1 (RMSD = 1.076), and VZV (RMSD = 2.097) (Figure 2C). The results demonstrated a high degree of structural conservation between DEV *UL50* protein and its homologues in other *α*-herpesviruses members, suggesting a potentially conserved functional role across these viruses. SignalP 6.0 analysis indicated the absence of a signal peptide or transmembrane domain (Figure S1). Subcellular localisation prediction using DeepLoc suggested distribution in both the nucleus (score = 0.6245) and cytoplasm (score = 0.6148) (Figure 2D). Consistently, immunofluorescence assays confirmed that *UL50* protein was present in both cellular compartments during infection, with stronger fluorescence observed in the nucleus (Figure 2E). These findings collectively define the molecular characteristics, evolutionary conservation, and predicted cellular localisation of the DEV *UL50* protein.

### 3.2. Construction of *UL50* gene-deleted virus and its repaired strain

**Figure 3.**
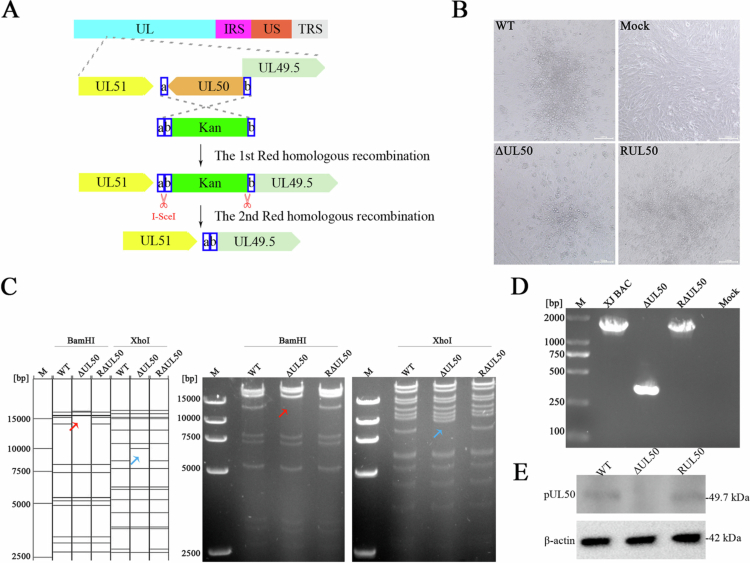
Construction of *UL50* gene-deleted virus and its repaired strain. (A) Schematic representation of the two step red-mediated recombination to process used to construct the ΔUL50 strain. The *UL50* genomic region and its flanking genes (*UL49.5* and *UL51*) are shown. The entire UL50 open reading frame was deleted. (B) The BAC-free *UL50*-deleted strain (ΔUL50), *UL50*-repaired strain (RUL50), and parental strain (WT) were harvested following rescue and purification. (C) RFLP analysis of pXJ-BAC-ΔUL50 and pXJ BAC-RUL50. The left panel shows the predicted patterns generated using Vector NTI, while the right panel presents the experimental results. Red arrows indicate differences among recombinant viruses observed after BamHI digestion, while blue arrows highlight variations following XhoI digestion. (D) PCR analysis confirming the identity of recombinant viruses. (E) Western blot analysis verifying the expression of the DEV *UL50* protein. Cell lysates from recombinant virus-infected cells were subjected to Western blotting and probed with anti-UL50 polyclonal antibody. *β*-actin served as a loading control.

The *UL50* gene, which encodes a non-structural protein of DEV, was selected for genetic analysis. A scarless *UL50* gene-deleted strain (ΔUL50) and its repaired strain (RUL50) were generated using a two-step Red recombination strategy based on the DEV BAC system (Figure 3A and B). Positive BAC clones were confirmed via restriction fragment length polymorphism (RFLP) analysis (Figure 3C). DNA from selected colonies was extracted and digested with *BamH*I and *Xho*I, with the results showing complete concordance between the observed band patterns and the predicted results. Recombinant virus ΔUL50 and RUL50 were identified through PCR amplification using *UL50*-specific primers (UL50 F/R). The amplification produced a 1707 bp fragment in both the wild-type (WT) and RUL50 strain, consistent with the intact *UL50* gene, whereas a 363 bp fragment was observed in the ΔUL50 strain, confirming successful deletion of *UL50 gene* (Figure 3D). To assess *UL50* protein expression, DEFs infected with the recombinant viruses were analysed by Western blot using rabbit polyclonal antibodies specific to UL50 protein, *β*-actin served as a loading control (Figure 3E). No *UL50* protein was detected in ΔUL50-infected cells, confirming successful deletion at the protein level. To evaluate the genetic stability of the recombinant virus, ΔUL50 was continuously passaged in DEFs, and PCR analysis of passages F5, F10, F15, and F20 showed no evidence of *UL50* reversion (Figure S2A). Furthermore, viral titer measurements across these passages revealed no significant differences between generations (Figure S2B), indicating that the ΔUL50 maintained stable titre throughout serial passaging.

### 3.3. Growth characteristics of the *UL50* gene-deleted virus in DEFs

**Figure 4.**
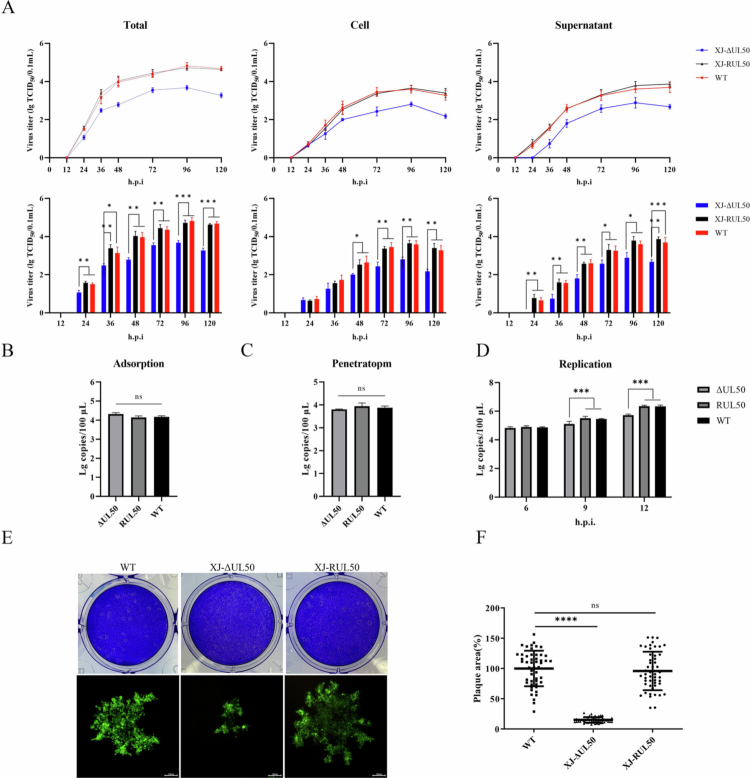
Comparison of biological characteristics among ΔUL50, RUL50, and WT strain. (A) Multistep growth kinetics of recombinant viruses. Viral titre in infected cells were measured at different time points following inoculation with approximately 0.02 MOI of virus. Statistical analyses were performed on the total, cell, and supernatant viral titre at each time point. Growth curves represent the mean values from three independent experiments. (B) Viral adsorption assays. DEFs were infected with the indicated viruses at 4 °C, and viral DNA copy numbers were quantified from collected samples. (C) Viral penetration assays. Following adsorption, DEFs were incubated at 37 °C for 1 hour, and viral DNA copy numbers were quantified from collected cell samples. (D) Viral replication assay. Cell samples were collected at 6, 9, and 12 hours post-infection, and viral DNA copy numbers were determined. (E) Plaque morphology of recombinant viruses. Upper panel: DEFs were infected with WT, ΔUL50, and RUL50 at the same MOI and stained with crystal violet at 6 days post-infection. Lower panel: Recombinant viruses carrying a GFP reporter gene were used to further visualise plaque formation. (F) Plaque sizes were measured using ImageJ software. A total of 50 plaques per sample were analysed for quantification (ns, *P* > 0.05; *, *P* < 0.05; **, *P* < 0.01; ***, *P* < 0.001; ****, *P* < 0.0001).

To investigate the growth kinetics of the recombinant viruses, multistep growth patterns of the WT, ΔUL50, and RUL50 strains were compared *in vitro* (Figure 4A). All three viruses exhibited similar overall replication kinetics, characterised by a rapid increase in viral titre during the early phase of infection and reaching peak levels at 96 h post-infection, followed by a plateau or slight decline thereafter. Specifically, ΔUL50 propagated to significantly lower viral titre than the WT and RUL50 strains in total virus, supernatant, and intracellular fractions at multiple time points. At the peak of replication (96 h post-infection), the total viral titer of ΔUL50 was approximately 1 log lower than that of the WT and RUL50 strains. Statistical analysis confirmed that these differences were significant across most time points examined (*P* < 0.001). In contrast, the RUL50 strain exhibited replication kinetics and viral titre nearly identical to those of the WT virus, indicating that reintroduction of *UL50* fully restored viral replication in DEFs. Furthermore, our previous study demonstrated that BAC insertion does not affect the replication of DEV in DEFs (Huo et al. 2024). Consistently, BAC insertion also had no impact on the replication of the ΔUL50 and RUL50 strains in DEFs (Figure S3).

To further explore the potential role of the *UL50* gene during the early stages of the viral infection, the viral adsorption, penetration, and replication assays were conducted using qPCR. The results showed no significant differences in viral DNA copy numbers among the WT, ΔUL50, and RUL50 groups during the adsorption and penetration phases (Figure 4B and C). However, during the replication phase, intracellular viral DNA copy number in the ΔUL50 group was significantly lower at 9 and 12 h post-infection compared to the WT and RUL50 strains (Figure 4D). These findings suggest that the deletion of the *UL50* gene impairs viral replication during the early stages of infection. These data indicate that deletion of *UL50* affects the efficiency of viral genome amplification in DEFs but does not impair initial viral entry processes.

The viral plaque morphometry of the WT, ΔUL50, and RUL50 strains was compared *in vitro* at an infection dose of 0.02 MOI (Figure 4E). Statistical analysis using imageJ and GraphPad Prism 9.4.0 revealed that the plaque areas were significantly reduced in ΔUL50-infected cells compared to the WT-infected cells (*P* < 0.0001), with plaques being approximately 84%–85% smaller than in the other two groups (Figure 4F). However, no significant differences were observed between RUL50 and WT groups. To further visualise plaque morphology differences, a fluorescence-based plaque assay was performed, which confirmed the significantly reduced plaque size of the ΔUL50 mutant. The absence of *UL50* impairs viral plaque formation, indicating its important role in the viral replication process.

### 3.4. Effect of *UL50* gene deletion on pathogenesis and mortality in ducks

**Figure 5.**
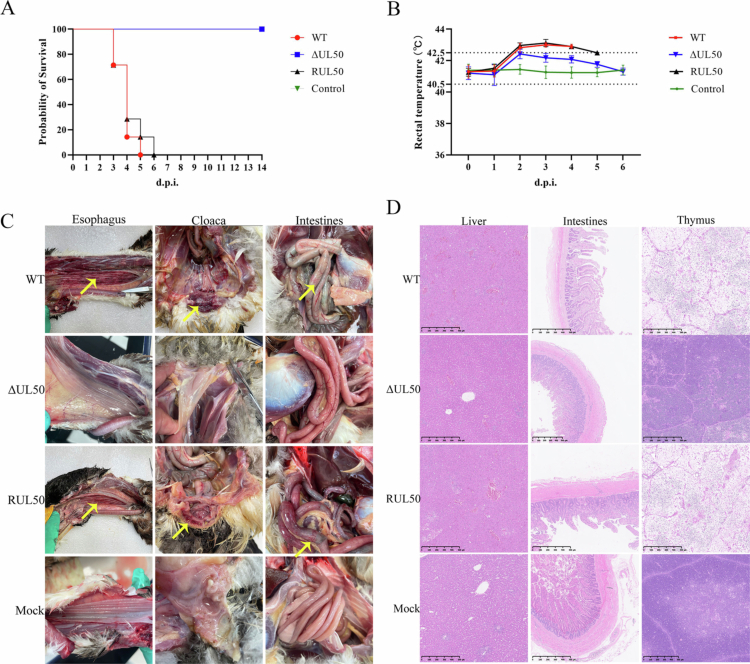
Pathogenesis of ΔUL50, RUL50, and WT strain. (A) Survival curve. Ducks (*n* = 10 per group) were infected with ΔUL50, RUL50, or WT strains, with DMEM used as a negative control. Survival rates were monitored over time. (B) Rectal temperature. The rectal temperatures of infected ducks were measured daily for 10 days post-infection, and temperature trends for each group were plotted. (C) Histopathological analysis. Autopsy findings from infected ducks. Yellow arrows indicate organ lesions. In the WT and RUL50 groups, pronounced haemorrhagic spots were observed in the oesophagus and cloaca, along with circular haemorrhagic spots on the intestinal surface. In contrast, no lesions were detected in the ΔUL50 group or the negative control group. (D) Microscopic observation of visceral lesions in ducks post-infection. Sheldrake, liver, intestines and thymus, HE staining, 1 bar = 5 μm. In the liver of the WT and RΔUL50 groups, significant blood cell accumulation, pronounced haemorrhage, and cellular degeneration and necrosis were observed. Large areas of epithelial cells from the intestinal villi were detached and released into the intestinal lumen. Thymic lobules showed indistinct boundaries, with necrosis of the medullary reticular cells and cortical lymphocyte. In contrast, the histopathological changes of liver, intestine, and thymus in the ΔUL50 group were significantly alleviated.

To evaluate whether deletion of the *UL50* gene alters the lethality and pathogenesis of DEV infection, 60-day-old ducks confirmed to be free of DEV and anti-DEV antibodies were intramuscularly injected with liver homogenate supernatant of WT, ΔUL50, and RUL50 strains. The mortality curve revealed that ducks infected with RUL50 and WT strains exhibited a similar mortality pattern (Figure 5A). Mortality began on the third day post-infection, followed by a rapid increase from the fourth to fifth day, reaching 100% by the fifth or sixth day post-infection. In contrast, all ducks infected with the ΔUL50 strain survived throughout the 10-day observation period.

Rectal temperatures of ducks were monitored daily. The results showed that the RUL50 and WT groups exhibited a similar trend, with temperatures rising on the second day post-infection and peaking on the third day (Figure 5B). The highest rectal temperature recorded was 43.1 °C in the RUL50 group and 42.9 °C in the WT group. In contrast, the ΔUL50 group maintained rectal temperatures within the normal range (40.5~42.5 °C) throughout the 6-day observation period, despite a slight increase on the second day post-infection.

### 3.5. Effect of *UL50* deletion on DEV-induced systemic tissue damage and histopathological alterations

To further investigate the pathogenesis of DP, ducks that died during the experiment were dissected, and the pathological lesions were observed and recorded (Figure 5C). Autopsy results revealed that in the WT and RUL50 groups, obvious haemorrhagic spots were observed in the oesophagus and cloaca, with circular haemorrhagic lesions visible on the surface of the intestines. No macroscopic lesions were observed in the negative control and ΔUL50 groups.

The liver, intestines, and thymus were collected and fixed in 4% paraformaldehyde for histopathological examination (Figure 5D). Microscopic analysis of visceral lesions showed that in the liver of the WT and RUL50 groups, there was significant blood cell accumulation, evident haemorrhage, and cellular degeneration and necrosis. Large areas of intestinal villi were detached, with epithelial cells released into the intestinal lumen. Thymic lobules exhibited poorly defined boundaries, with necrosis of medullary reticular cells and cortical lymphocytes. In contrast, the histopathological changes of liver, intestine, and thymus in the ΔUL50 group were significantly alleviated. Overall, the ΔUL50 strain induced markedly reduced gross and microscopic tissue damage compared with WT and RUL50, indicating that deletion of *UL50* significantly attenuates DEV-associated pathological changes *in vivo*.

### 3.6. Effect of *UL50* gene deletion on viral replication in ducks

**Figure 6.**
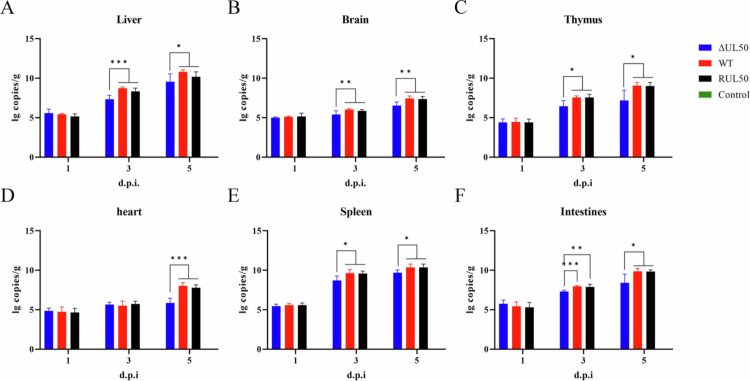
Replication characteristics of recombinant virus *in vivo*. Tissue samples were collected from infected ducks at indicated time points post-infection to assess viral replication by qPCR. DEV viral load were quantified in the liver (A), brain (B), Thymus (C), heart (D), spleen (E), and intestines (F) (*n* = 5;ns, *P *> 0.05;*, *P *< 0.05**;**, *P *< 0.01;***, *P *< 0.001).

The tissue organs of infected ducks were collected at various time points post-infection from each group to assess *in vivo* viral replication by qPCR (Figure 6). The viral loads in the liver, brain, intestine, heart, kidneys, and thymus of the WT, ΔUL50, and RUL50 groups all showed an increasing trend after virus infection. No significant differences were observed in the viral loads between the RUL50 and WT groups. In contrast, viral loads in the organs of the ΔUL50 group increased at a slower rate. Statistical analysis revealed a significant reduction in the viral DNA copy number in these tissues following infection with the ΔUL50 strain compared to the WT and RUL50 strains. These results indicate that although deletion of *UL50* does not eliminate viral replication *in vivo*, it markedly reduces the replication efficiency of DEV in multiple tissues. The diminished viral replication observed in ducks infected with ΔUL50 correlates with the attenuated tissue damage, reduced clinical manifestations, and the absence of mortality.

### 3.7. Replication of the *UL50* gene-deleted virus in DRG neurons

**Figure 7.**
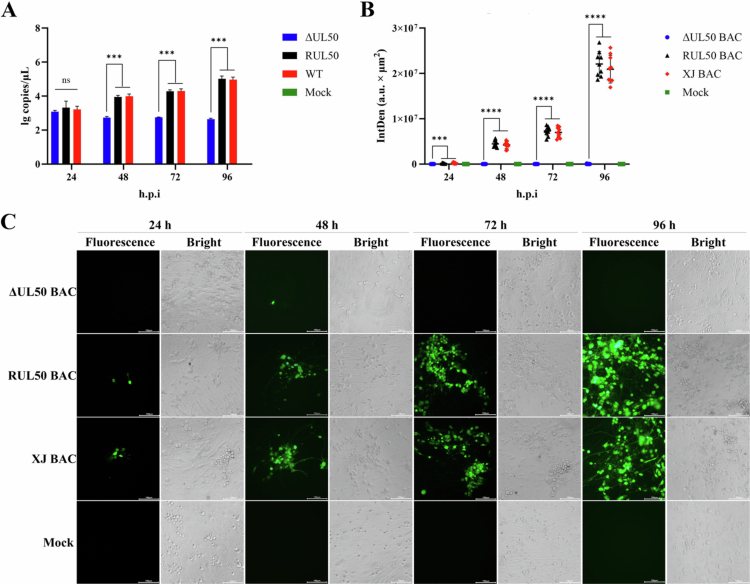
Replication of recombinant viruses in DRG neurons. (A) Viral replication assay. DRG neurons were infected with ΔUL50, RUL50, and WT at an MOI of 0.1. Samples were collected at 24, 48, 72, and 96 hpi, and viral DNA copy numbers were quantified by qPCR. (B) Fluorescence intensity analysis. DRG neurons were infected with ΔUL50 BAC, RUL50 BAC, and XJ BAC (each carrying a GFP reporter gene) at an MOI of 0.1. Fluorescence intensity was quantified by randomly selecting multiple fields of view using ImageJ. (C) Viral spread assessment. EGFP expression was used to monitor the proliferation of recombinant viruses in DRG neurons. (ns, *P* > 0.05; ***, *P* < 0.001; ****, *P* < 0.0001).

**Figure 8.**
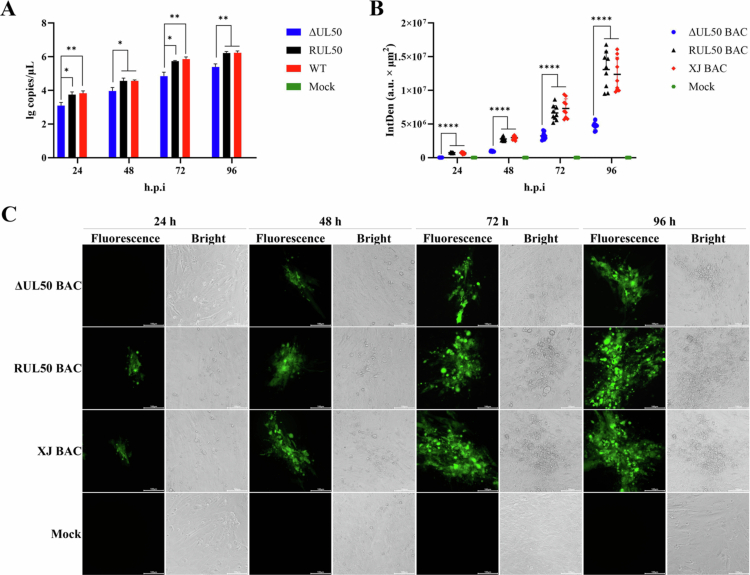
Replication of recombinant viruses in DEFs. (A) Viral replication assay. DEFs were infected with ΔUL50, RUL50, and WT at an MOI of 0.1, simultaneously with DRG neurons. Samples were collected at 24, 48, 72, and 96 hpi, and viral DNA copy numbers were quantified by qPCR. (B) Fluorescence intensity analysis. DEFs were infected with ΔUL50 BAC, RUL50 BAC, and XJ BAC at an MOI of 0.1, simultaneously with DRG neurons. Fluorescence intensity was analysed by randomly selecting multiple fields of view using ImageJ. (C) Viral spread assessment. EGFP expression was used to monitor the proliferation of recombinant viruses in DEFs. (ns, *P* > 0.05; *, *P* < 0.05; **, *P* < 0.01; ****, *P* < 0.0001).

Given the marked reduction (*P* < 0.05) of viral loads in brain tissues observed *in vivo*, we next examined viral replication in primary duck DRG neurons. Given that neuronal cells are terminally differentiated and non-dividing, this observation prompted us to further examine whether *UL50* deletion differentially affects viral replication in neuronal cells. Therefore, we compared the replication properties of the parental, ΔUL50, and revertant viruses in primary duck dorsal root ganglion (DRG) neurons and, for comparison, in DEFs. Viral DNA copy numbers were quantified by qPCR at 24, 48, 72 and 96 hpi. The results indicated that ΔUL50 exhibited almost no replication in DRG neurons (Figure 7A). In contrast, while viral DNA copy numbers in the ΔUL50 group were significantly lower than those in the WT and RUL50 groups at each time point, the virus was still able to replicate in DEFs (Figure 8A). Furthermore, DRG neurons and DEFs were infected with ΔUL50 BAC, RUL50 BAC and XJ BAC, all of which carry a GFP reporter gene. Fluorescence microscopy images were analysed by randomly selecting multiple fields of view using ImageJ. The results revealed that no spread of ΔUL50 BAC was observed in primary DRG neurons (Figure 7B and C). In DEFs, although the fluorescence intensity in the ΔUL50 BAC group was significantly lower than that in the RUL50 BAC and XJ BAC groups at each time point, ΔUL50 BAC was still capable of spreading within DEFs (Figure 8B and C). Collectively, these findings indicate that *UL50* deletion substantially restricts viral replication and cell-to-cell spread in primary DRG neurons, whereas replication remains detectable—though reduced—in dividing DEFs.

## 4. Discussion

Duck plague (DP) is a highly infectious disease that poses a serious threat to waterfowl farming. Although DEV virulence and vaccine development have been studied for decades (Tian et al. 2019; Ning et al. 2024; Jia et al. 2025), progress in understanding its molecular pathogenesis has lagged behind that of other herpesviruses, partly due to the genomic complexity of DEV, limited antibody resources, and challenges in cell culture adaptation. Previous studies have identified several genes such as TK, US10, and the gI/gE complex are dispensable for replication but are closely associated with virulence (Li et al. 2006; Liu et al. 2020). In this study, we focused on *UL50*, a DEV gene previously annotated to encode a dUTPase-like protein based on conserved motifs shared with *α*-herpesvirus homologues (Ariza et al. 2022). Bioinformatic analysis revealed that DEV *UL50* retains typical structural features and conserved domains of this protein family. The absence of signal peptides and transmembrane regions indicates that *UL50* is a soluble intracellular protein, while both prediction tools and IFA assays identified its presence in the cytoplasm and nucleus. This localisation pattern is consistent with the general role of early herpesvirus genes during the replication cycle. Importantly, deletion of *UL50* resulted in reduced replication efficiency, supporting that this gene contributes positively to DEV replication.

In mammalian *α*-herpesviruses such as HSV-1 and PRV, *UL50* homologues are similarly non-essential for replication but contribute to virulence and immunogenicity (Pyles et al. 1992; Jons et al. 1997). Consistent with these findings, ΔUL50-infected ducks in our study showed no mortality and only mild transient fever, indicating attenuation of DEV upon *UL50* deletion. Taken together with our sequence and structural analyses, which revealed conserved catalytic motifs and domains shared among *α*-herpesvirus *UL50* homologues, these findings support the notion that *UL50* serves a functionally conserved role across this viral subfamily. This highlights its contribution not only to viral replication efficiency but also to pathogenesis, reinforcing its relevance in the broader context of *α*-herpesvirus biology.

A notable finding of this study was although ΔUL50 retained replication capacity in proliferating DEFs, viral loads were markedly reduced in infected ducks, with the pronounced decrease observed in brain tissues. This phenotype was further confirmed in primary duck DRG neurons, where ΔUL50 replication was nearly abolished. Similar cell-type–dependent effects have been reported in other viruses such as HSV-1, african swine fever virus and D-type retroviruses, in which replication remains largely intact in dividing cells but is impaired in non-dividing cells (Pyles et al. 1992; Oliveros et al. 1999; Payne and Elder 2001). These findings indicate that the viral dUTPase encoded by *UL50* plays a critical role in supporting DEV replication in non-dividing cells, which may relate to the neurotropism of DEV and likely contributes to the attenuated pathogenicity observed upon *UL50* deletion.

In addition to its role in replication, the unique motif VI found between conserved motifs II and IV—a feature shared by herpesvirus *UL50* homologues—suggests that *UL50* may possess viral functions beyond basic nucleotide-related pathways (McGeehan et al. 2001). Prior research in other viruses has proposed that similar proteins may participate in immunomodulatory processes or host-pathway interactions (Padgett et al. 2004; Glaser et al. 2005; Waldman et al. 2008; Ariza et al. 2014; Williams et al. 2019; Cox et al. 2022). While the present study confirmed that *UL50* affects replication efficiency and virulence, the precise mechanisms remain unclear. Whether its effects are mediated through interactions with host pathways, modulation of innate immunity, or other regulatory functions warrants further investigation.

Deletion of *UL50* also resulted in a clearly attenuated phenotype *in vivo*. No mortality was observed in safety experiment (Table S1), although a transient mild fever occurred following immunisation (Figure S4A). Moreover, ΔUL50-immunised ducks were fully protected against lethal challenge under the experimental conditions tested (Figure S4B). After challenge, rectal temperatures and cloacal swab viral loads in ΔUL50-immunised birds were comparable to those of ducks vaccinated with a commercial vaccine (Figure S4C and S4D). These findings indicate that *UL50* deletion confers a genetically defined attenuation phenotype while retaining protective immunogenicity in a standardised challenge model. Consistent with the established strategy of attenuating herpesviruses through targeted deletion of non-essential virulence-associated genes, such as US3, gC/gE, and ICP27 (Ruan et al. 2022; Wei 2023; Wu et al. 2023), *UL50* deletion similarly yields a rationally attenuated mutant. However, given the reduced replication efficiency and the transient post-immunisation fever observed, further optimisation would be required before considering practical application. Comprehensive evaluation of safety, genetic stability, and long-term immune durability will also be essential. Therefore, ΔUL50 should be regarded as a functionally attenuated mutant that provides mechanistic insight into DEV virulence and may inform future rational vaccine design efforts.

In conclusion, this study provides a systematic functional characterisation of the DEV *UL50* gene, addressing a major gap in waterfowl herpesvirus research. We demonstrate that *UL50* encodes a conserved intracellular protein that enhances viral replication and contributes to DEV pathogenicity, particularly in non-dividing neuronal cells. The attenuation observed upon *UL50* deletion highlights its role as a virulence-associated gene and refines our understanding of DEV biology. These findings establish a framework for future investigations into UL50-mediated host–virus interactions and rational attenuation strategies in waterfowl herpesviruses.

## Supplementary Material

Supplementary_Materialclean.docx Supplementary_Materialclean.docx

## Data Availability

All the data generated during this study are included in the manuscript. Additional data related to this article may be requested from the corresponding authors.
