# Supplementary material for: Functional characterization of UL50 gene reveals its essential role in duck enteritis virus replication and pathogenesis
Source: Vet Q. 2026 Mar 24;46(1):2649575. doi: 10.1080/01652176.2026.2649575 (PMC13015092; doi:10.1080/01652176.2026.2649575)
Supplement: Supplementary_Materialclean.docx [file TVEQ_A_2649575_SM5229.docx]

**Supplementary Information for**

**Functional Characterization of UL50 Gene Reveals Its Essential Role in Duck Enteritis Virus Replication and Pathogenesis**

**Method**

**Genetic stability of XJ BAC in vitro.** DEFs cells were separately infected with different passages of ΔUL50, and viral harvests were collected 72 hour post-infection. Samples were processed with a Tissuelyser-24 at 60 Hz for 90 s and centrifuged at 5000 rpm before titration. Viral titers were determined by calculating the 50% tissue culture infectious dose (TCID50), and statistical analyses were conducted using GraphPad Prism version 9.4.0. All experiments were performed and repeated in triplicate.

**The immune protection effect of recombinant virus on ducks.** To evaluate whether the ΔUL50 strain can effectively protect ducks from lethal challenge by a virulent DEV strain, a safety assay was first conducted. 30-day-old ducks, confirmed to be free of DEV and DEV antibodies, were intramuscularly injected with ΔUL50 at doses of 10^3^ TCID_50_ (n=10), 10^4^ TCID_50_ (n=10), and 10^5^ TCID_50_ (n=10), along with a DMEM control groups (n=10). The ducks were monitored daily for body temperature and mortality. Subsequently, 30-day-old ducks, also confirmed to be free of DEV and DEV antibodies, were intramuscularly immunized with ΔUL50 at 10^4^ TCID_50_ (n=10), the same dose of a commercial attenuated vaccine (n=10), and DMEM (n=10). 14 days post-immunization, the ducks were challenged with 100 LD_50_ of the virulent DEV strain CV. Body temperature and mortality were recorded, and anal swab were collected to assess the viral shedding.

| **Table S1.** Survival statistics of ducks | |
| --- | --- |
| **Group** | **Survival** |
| 10^3^ TCID_50_ | 10/10 |
| 10^4^ TCID_50_ | 10/10 |
| 10^5^ TCID_50_ | 10/10 |
| Control | 10/10 |


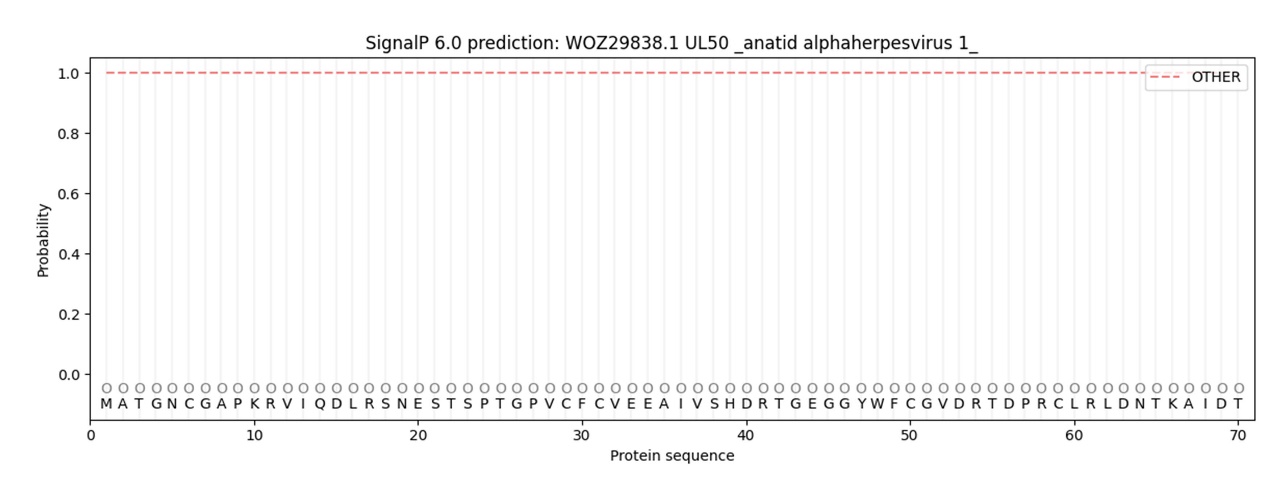


**Figure S1. Signal peptide analysis of the DEV UL50-encoded protein.** Prediction using SignalP 6.0 showed that the UL50-encoded protein lacks a classical signal peptide, consistent with its characterization as an intracellular viral protein.

**
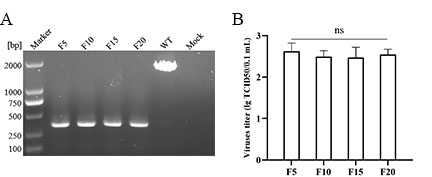
**

**Figure S2. Genetic stability of recombinant virus ΔUL50.** (A) PCR detection of *UL50* gene deletion in passages F5, F10, F15, and F20. The amplification product of the WT strain was 1707 bp, while that of the ΔUL50 strain was 363 bp. (B) The viral titers of ΔUL50 passages F5, F10, F15, and F20 were determined, with no significant differences observed among the four passages (ns, P > 0.05).

**
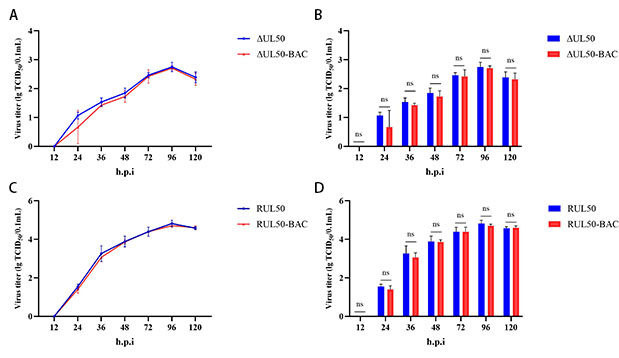
**

**Figure S3. Multistep growth kinetics of recombinant viruses.** Viral titers were measured at various time points after infection with approximately 0.02 MOI of virus. Statistical analysis of viral titers at each time point is shown in panels B and D (ns, P > 0.05). Growth curves in panels A and C represent the mean values from three independent experiments.

**
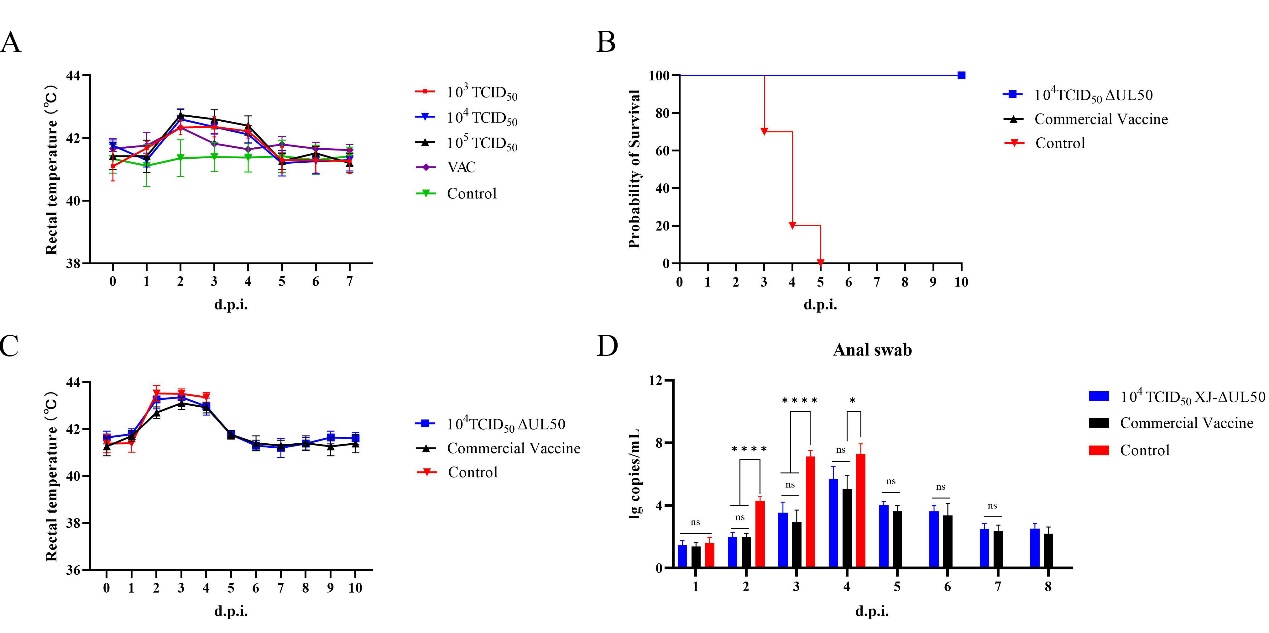
Figure S4. Safety and challenge protection assay of ΔUL50 strain.** (A) Rectal temperatures of immunized ducks (n = 10 per group) were measured daily for 7 days post-infection, and temperature trends were plotted for each group. (B) Survival curve. Immunized ducks (n=10 per group) were challenged with 100 LD_50_ of DEV virulent strain, and survival rates were monitored over time. (C) Rectal temperatures of challenged ducks (n = 5 per group) were measured daily for 10 days post-infection, and temperature trends were plotted. (D) Viral load in cloacal swab. DNA copy numbers were quantified from cloacal swabs collected from challenged ducks (n = 5; ns, *P* > 0.05; *, *P* < 0.05; ****, *P* < 0.0001).
